# Genomic regions associated with pseudorabies virus infection status in naturally infected feral swine (*Sus scrofa*)

**DOI:** 10.3389/fgene.2023.1292671

**Published:** 2023-11-23

**Authors:** Courtney F. Bowden, Jennifer N. Kiser, Ryan S. Miller, Alexandra C. Buckley, Paola M. Boggiatto, Rachael M. Giglio, Vienna R. Brown, Dorian Garrick, Holly L. Neibergs, Antoinette J. Piaggio, Scott E. Speidel, Timothy J. Smyser

**Affiliations:** ^1^ National Wildlife Research Center, Wildlife Services, Animal and Plant Health Inspection Service, United States Department of Agriculture, Fort Collins, CO, United States; ^2^ Department of Animal Sciences, College of Agricultural, Human, and Natural Resource Sciences, Washington State University, Pullman, WA, United States; ^3^ Center for Epidemiology and Animal Health, Veterinary Services, Animal and Plant Health Inspection Service, United States Department of Agriculture, Fort Collins, CO, United States; ^4^ Virus and Prion Research Unit, National Animal Disease Center, Agricultural Research Service, United States Department of Agriculture, Ames, IA, United States; ^5^ Infectious Bacterial Diseases Research Unit, National Animal Disease Center, Agricultural Research Service, United States Department of Agriculture, Ames, IA, United States; ^6^ National Feral Swine Damage Management Program, Wildlife Services, Animal and Plant Health Inspection Service, United States Department of Agriculture, Fort Collins, CO, United States; ^7^ AL Rae Centre for Genetics and Breeding, Massey University, Palmerston North, New Zealand; ^8^ Department of Animal Sciences, College of Agricultural Sciences, Colorado State University, Fort Collins, CO, United States

**Keywords:** Aujeszky’s disease, disease spillover, feral swine, GSEA-SNP, GWAS, pseudorabies virus (PRV), pseudo-heritability

## Abstract

Pseudorabies virus (PRV)—the causative agent of Aujeszky’s disease—was eliminated from commercial pig production herds in the United States (US) in 2004; however, PRV remains endemic among invasive feral swine (*Sus scrofa*). The circulation of PRV among abundant, widespread feral swine populations poses a sustained risk for disease spillover to production herds. Risk–based surveillance has been successfully implemented for PRV in feral swine populations in the US. However, understanding the role of host genetics in infection status may offer new insights into the epidemiology and disease dynamics of PRV that can be applied to management strategies. Genetic mechanisms underlying host susceptibility to PRV are relatively unknown; therefore, we sought to identify genomic regions associated with PRV infection status among naturally infected feral swine using genome–wide association studies (GWAS) and gene set enrichment analysis of single nucleotide polymorphism data (GSEA–SNP). Paired serological and genotypic data were collected from 6,081 feral swine distributed across the invaded range within the contiguous US. Three complementary study populations were developed for GWAS: 1) comprehensive population consisting of feral swine throughout the invaded range within the contiguous US; 2) population of feral swine under high, but temporally variable PRV infection pressure; and 3) population of feral swine under temporally stable, high PRV infection pressure. We identified one intronic SNP associated with PRV infection status within candidate gene *AKAP6* on autosome 7. Various gene sets linked to metabolic pathways were enriched in the GSEA–SNP. Ultimately, improving disease surveillance efforts in feral swine will be critical to further understanding of the role host genetics play in PRV infection status, helping secure the health of commercial pork production.

## 1 Introduction

Pseudorabies virus (PRV), also referred to as suid herpesvirus 1 (SuHV–1), is a DNA herpesvirus in the Herpesviridae family (Mettenleiter et al., 2019). Members of the Suidae family are the only natural hosts for PRV; however, numerous mammals can be naturally or experimentally infected with PRV (Mettenleiter, 2008; Bo and Li, 2022). The primary route of transmission among swine (*Sus scrofa*) is direct oronasal contact; however, venereal, aerosol, transplacental, and fomite–mediated transmission can also occur (Mettenleiter, 2008; Mettenleiter et al., 2019; Bo and Li, 2022). In swine, the virus primarily replicates in the epithelial cells of the upper respiratory tract prior to reaching the tonsils and local lymph nodes. Pseudorabies virus can gain access to neurons innervating the facial and oropharyngeal region and ultimately spread to the cell bodies of infected neurons, where a lytic or latent infection occurs (Verpoest et al., 2017; Mettenleiter et al., 2019). Strains of PRV differ in virulence, which impacts both clinical disease and tissue tropism. Highly virulent strains of PRV primarily cause neuroinvasion, whereas strains of PRV with low to moderate virulence have weak neuroinvasiveness but a distinct tropism for the respiratory tract. Highly adapted and/or attenuated strains often demonstrate a tropism for the reproductive tract (Mettenleiter et al., 2019).

Clinical manifestation of disease following PRV infection is highly dependent upon the age of the affected swine (Nauwynck, 1997; Mettenleiter, 2008; Verpoest et al., 2017). Piglets, less than 3 weeks of age, are highly susceptible to PRV with up to 100% mortality and may exhibit high fever, dullness, anorexia, vomiting, weakness, incoordination, twitching, and convulsions. As swine surpass 3 weeks of age, neurological symptoms are less common, as is mortality. Mature pigs primarily exhibit respiratory distress in the form of coughing, sneezing, and heavy breathing. If gestational infection occurs, fetal resorption, mummification, abortions, or weakened piglets may be observed. Individuals that recover from clinical infection can remain latently infected in their olfactory bulb, trigeminal ganglia, brain stem, or sacral ganglia (Mettenleiter, 2008; Verpoest et al., 2017; Mettenleiter et al., 2019; USDA APHIS, 2020a). At the herd level, PRV results in morbidity and mortality of afflicted individuals and cumulative production losses (Anderson et al., 2008).

In the United States (US), invasive populations of feral swine (*Sus scrofa*) are known reservoirs for PRV and pose a sustained risk of disease spillover for domestic herds, from which PRV was eliminated in 2004 (Anderson et al., 2008; Pedersen et al., 2013; Hernández et al., 2018b). Free–ranging feral swine populations have been present in the US since domestic pigs were first introduced in 1539 to the Florida peninsula. Historically, feral swine populations were composed of domestic pigs that were either released or escaped as a consequence of free–range livestock practices; however, contemporary populations generally represent animals of mixed wild boar and domestic pig ancestry following the importation of European wild boar for hunting purposes (Smyser et al., 2020). Despite their long history in the US, the distribution of feral swine has expanded dramatically in recent years largely due to human-facilitated movement (i.e., translocation; Bevins et al., 2014; McClure et al., 2015; Snow et al., 2017; Tabak et al., 2017; Hernández et al., 2018a; Smyser et al., 2020). This expansion increases the risk of disease spillover, as feral swine are now established in agricultural productions regions with domestic livestock, poultry, and cervids that demonstrate susceptibility to feral swine pathogens (Miller et al., 2017). Miller et al. (2017) found that in 2012, an average of 47.7% of all farms in the contiguous US were in counties with known feral swine populations. Furthermore, previous studies have reported the presence of feral swine in livestock housing areas (Anderson et al., 2019) and documented feral swine consuming livestock feed and supplements (Carlisle et al., 2021). Feral swine have also been observed in close proximity to pork production facilities (Wyckoff et al., 2009; Engeman et al., 2011) including outdoor operations (Patterson et al., 2022). Such interactions are particularly worrisome given that PRV is primarily spread via direct contact between animals (Mettenleiter et al., 2019; USDA APHIS, 2020b).

Eliminating PRV from feral swine in the US is unlikely due to the abundant and widespread nature of invasive populations as well as the high prevalence of PRV, with apparent seroprevalence of 22% across the invaded range (USDA APHIS WS unpublished data, 2020). Additionally, traditional methods of PRV mitigation among domestic herds (e.g., depopulation or vaccination; Anderson et al., 2008) cannot be readily applied to this free–ranging and prolific invasive species. Thus, a logical approach for controlling PRV in feral swine is using disease surveillance data to quantify pathogen transmission on the landscape and ensuring that allocated control resources are commensurate with associated disease spillover risks (Müller et al., 2011; Brown et al., 2019). Targeted, risk–based surveillance has been successfully implemented for PRV across feral swine populations throughout the US (Brown et al., 2019; Brown et al., 2020). The National Feral Swine Damage Management Program (NFSP), a United States Department of Agriculture (USDA) program that facilitates feral swine control efforts throughout the invaded range and collects associated biological samples for disease surveillance, screens approximately 6,000 feral swine annually for diseases of national concern. However, understanding the role of host genetics in susceptibility may offer new insights into the epidemiology and disease dynamics of PRV that can be applied to management strategies (Robinson et al., 2012; Blanchong et al., 2016; Queirós et al., 2018; Seabury et al., 2020).

Accordingly, our goal was to utilize serological data collected under USDA disease surveillance efforts and paired single nucleotide polymorphism (SNP) genotypes to build upon previous studies that have identified loci associated with susceptibility/resistance to PRV in experimentally and naturally infected domestic pigs (Reiner et al., 2002; Yuan et al., 2009) and European wild boar (Fabbri et al., 2021). Specifically, our objective was to 1) identify loci associated with PRV infection status among naturally infected feral swine sampled throughout their invaded range within the contiguous US using case/control genome–wide association studies (GWAS) and 2) use a pathway–based approach, gene set enrichment analysis of single nucleotide polymorphism data (GSEA–SNP), to analyze the GWAS data and identify gene sets (groups of genes that share biological function, regulation or chromosomal location; Subramanian et al., 2005) associated with PRV infection status.

## 2 Materials and methods

### 2.1 Sample collection

Blood, hair, and tissue samples were collected from adult feral swine (*n* = 6,081) throughout the invaded range within the contiguous US (33 states) as an extension of damage management efforts led by the United States Department of Agriculture (USDA) Animal and Plant Health Inspection Service (APHIS) Wildlife Services (WS). Samples were collected from May 2012 to September 2020 with the majority of samples (93%) collected since 2014. Samples were acquired ancillary to legally authorized control of invasive feral swine; therefore, sample collection was exempted from Institutional Animal Care and Use Committee review (Sikes et al., 2016).

### 2.2 Serological assay

Disease status was not known at the time of sampling, so cases and controls were not determined by study design. Individuals that were positive for PRV antibodies were considered cases, whereas individuals negative for PRV antibodies were considered controls.

Serum samples were tested for PRV antibodies at the Kentucky Federal Brucellosis Laboratory (Frankfort, Kentucky, United States) using the “short protocol” for Pseudorabies–gB Enzyme–Linked Immunosorbent Assay (PRV–gB ELISA; IDEXX Laboratories Inc., Westbrook, Maine, United States). Samples were considered positive for PRV if the S/N value ≤0.60 and negative if the S/N value >0.70. Individuals with 0.60 < S/N ≤ 0.70 were indeterminate in PRV infection status and were excluded from our analyses. Of the 6,081 samples tested for PRV antibodies, 1,294 were positive, and 4,787 were negative.

### 2.3 Genotype data

DNA extraction and genotyping were conducted at GeneSeek (Neogen Corporation, Lincoln, Nebraska, United States). DNA extraction was performed using the MagMAX™ DNA Multi-Sample Ultra Kit (Thermo Fisher Scientific Inc., Waltham, MA, United States) and genotyping was completed using the GeneSeek Genomic Profiler (GGP) for Porcine 80 k array [68,516 loci; Illumina BeadChip microarrays (San Diego, California, United States) licensed exclusive to GeneSeek, Neogen Corporation, (Lincoln, Nebraska, United States)]. Bi–allelic SNP were mapped to the Sscrofa11.1 reference genome assembly (Warr et al., 2020), and unmapped and non–autosomal markers were removed, leaving 62,128 loci for further consideration. Standard quality control filters for genotype data were implemented using SNP & Variation Suite (SVS; version 8.9.0; Golden Helix, Bozeman, Montana, United States), specifically removing samples with call rates <0.90 and then pruning loci with call rates <0.90 or minor allele frequency <0.05. Following quality control measures, we retained 5,875 feral swine samples (1,246 PRV positive and 4,629 PRV negative) and 56,024 loci for subsequent analyses.

### 2.4 Study populations

Imperfect diagnostics, incomplete pathogen exposure, and varying levels of infection pressure over time and space can result in biased, primarily lower, heritability and SNP effect estimates (Bishop and Woolliams, 2010; Bishop et al., 2012; Bishop and Woolliams, 2014). Therefore, from the 5,875 feral swine samples that passed genotype quality control, three study populations were derived based on the presence and persistence of PRV on the landscape.

#### 2.4.1 Comprehensive study population

The feral swine data were stratified into populations that experienced similar PRV exposure as well as abiotic and biotic factors that may interact with PRV to reduce fitness of infected individuals. We used watersheds as a means of defining populations and associated landscape scale features. Hydrologic units are a hierarchical classification system of watersheds that represent a discrete set of biotic and abiotic factors and serve as an ecologically relevant unit for aggregating landscape–level covariates (Miller, 2017; McClure et al., 2018). We chose the subbasin level [hydrological unit code (HUC) eight], here–after referred to as watershed, to encompass populations of feral swine experiencing similar environments, including PRV infection pressure (see description of true seroprevalence prediction below). Variation in PRV infection status amongst the samples was evaluated for each unique combination of sampling year and watershed; combinations that lacked variation in PRV infection status (i.e., all samples were either cases or controls) were excluded from the analysis. The remaining samples were considered the comprehensive study population during subsequent analysis.

#### 2.4.2 High PRV infection pressure with temporal variation

Feral swine samples were opportunistically collected during routine USDA APHIS Wildlife Services management efforts; therefore, misclassification of cases and controls could result from imperfect sero–diagnostics or a lack of PRV exposure on the landscape (Bishop et al., 2012). Serologic diagnostic assays can underestimate disease prevalence resulting in biased estimates of parameter effects and, in some cases, resulting in invalid inferences (Tabak et al., 2019). Therefore, true seroprevalence for PRV, that accounts for imperfect detection was predicted for each year in each watershed. Predictions were made using a hierarchical Bayesian model (see Supplementary Material) with 28,030 feral swine sampled across 37 states in the contiguous US between October 2010 and September 2020. Similar to the paired genetic and serological samples that serve as the focus of this study (*n* = 6,081), these samples were more broadly collected (temporally and spatially) by USDA APHIS Wildlife Services as a component of disease surveillance and population control efforts.

True seroprevalence can have year–to–year variation, and predictions are also influenced by the amount of data available to fit models. To reduce the likelihood of misclassifying an unexposed individual as a control, data were truncated to only include watersheds in which feral swine were sampled across ≥3 years. Watersheds were then partitioned into either high or low true seroprevalence groups using the median true seroprevalence (35.8%) observed across the comprehensive study population. Controls in the high true seroprevalence group were assumed to have experienced PRV infection pressure and thus were truly resistant to PRV. Accordingly, feral swine in the high true seroprevalence group, here–after referred to as the HTS study population, were retained for subsequent analysis.

#### 2.4.3 High PRV infection pressure without temporal variation

Changes in infection pressure can result from temporal variation; therefore, high and low true seroprevalence groups were further stratified as stable or unstable using the mean annual change in true seroprevalence (±14.8%) as a threshold. There is uncertainty associated with predictions of true seroprevalence; therefore, watersheds were identified as uncertain if the standard deviation of the true seroprevalence was greater than the mean annual change (±14.8%) and certain if the standard deviation was less. The certain, stable high true seroprevalence group, here–after referred to as the CSHTS study population, was retained for subsequent analysis.

### 2.5 Bioclimatic regions

Environmental conditions are known to influence PRV infection risk and may interact with PRV prevalence to influence selective pressures. Animals that experience physiological stress resulting from environmental conditions are more likely to have clinical manifestations of PRV that may reduce overall host fitness (Tozzini et al., 1982; Capua et al., 1997; de Groot et al., 2001). Accordingly, each watershed was assigned to a bioclimatic region using data from Metzger et al. (2013). Bioclimatic regions represent unique biological and climatic conditions and can be interpreted as a discrete representation of environmental gradients. The median number of watersheds assigned to a bioclimatic region across the Comprehensive, HTS, and CSHTS study populations were 21, 7.5, and 2, respectively.

### 2.6 Percent European wild boar ancestry

Feral swine in this study overwhelmingly represent individuals that are of mixed domestic swine and European wild boar ancestry. Management strategies of domestic livestock have reduced genetic diversity within host populations, which subsequently increases disease susceptibility (Springbett et al., 2003; King and Lively, 2012). In contrast, wild populations do not undergo genetic management or artificial selection for production traits but instead genomic processes are influenced by natural selective pressures including disease. Accordingly, one could hypothesize that individual feral swine with greater European wild boar ancestry would be more resilient to PRV.

The methods described in Smyser et al. (2020) were used to estimate the percent European wild boar ancestry of individual feral swine included in our analysis. Briefly, ADMIXTURE (Alexander et al. 2009) was used in a supervised framework to query an individual genotype against a comprehensive reference set for *Sus scrofa*, comprised of 105 domestic breeds, 23 wild boar populations, and 4 sister taxa. This method proportionately associates the origin of individual feral swine genotypes among the 17 ancestry groups that comprise the *Sus scrofa* wild–domestic species complex reference set.

### 2.7 Model selection

For each study population, binary logistic regression was used to examine the probability of an individual being PRV antibody positive given sex of the individual (male vs. female), percent European wild boar ancestry (continuous), PRV true seroprevalence (continuous), and bioclimatic region (6 unique bioclimatic regions for HTS and 5 unique bioclimatic regions for CSHTS). Since the effect of European wild boar ancestry may depend on infection pressure, an interaction between percent European wild boar ancestry and PRV true seroprevalence was also investigated. All predictors, including the interaction, were treated as fixed effects. Logistic regression models were fit using the R stats package (version 4.1.1; R Core Team, 2021).

Potential candidate models, representing all possible combinations of predictors, were evaluated using Akaike Information Criterion (AIC) implemented in R (version 4.1.1; R Core Team, 2021) package MuMIn (Bartoń, 2020). Akaike Information Criterion balances model parsimony with goodness of fit; the model with the lowest AIC value then represents the most informative or “best” model for predicting PRV infection status given the data and the candidate models considered (Bozdogan, 1987; Portet, 2020). To account for model uncertainty (i.e.*,* competing models within two Δ AIC of the “best” model), we examined the support for each predictor by calculating cumulative covariate weights where weights over 0.5 were considered supported (Doherty et al., 2012). Supported predictors were included as fixed effects in the mixed model GWAS described below.

### 2.8 Genome–wide association studies

Single–locus mixed model GWAS with efficient mixed–model association eXpedited (EMMAX) methodology (Kang et al., 2010) were performed for each study population using SNP & Variation Suite software (SVS; version 8.9.0; Golden Helix, Bozeman, Montana, United States). The mixed model equation can be expressed as:

y=Xβ+Zu+ε

where, y was an *n* × 1 vector of PRV infection status phenotypes, X was an *n* × *q* matrix of fixed effects (*q)*, **β** was an *q* × 1 vector of fixed effect coefficients to be estimated, **Z** was an *n* × *t* matrix relating random effects (*t*) to the phenotypes in y , u was a vector of random effects to be estimated, and **ε** was a vector of residuals. This model assumed that Var( u ) = $\sigma_{g}^{2}K$ and Var(ε) = $\sigma_{e}^{2}I$ ; thus, Var( y ) = $\sigma_{g}^{2}ZKZ^{\prime }+\sigma_{e}^{2}I$ . **K** was a pairwise genetic relatedness matrix and Z was the identity matrix I (Kang et al., 2008).

For each study population, three models of genetic architecture were evaluated to account for uncertainty regarding the mode of inheritance for PRV: additive, dominance, and recessive. Considering a bi-allelic SNP with alleles *D* and *d*, an additive model assumes a linear increase in disease risk with each additional copy of the risk allele (*D*); therefore, the risk for *DD* would be twice that of *Dd*. A dominance model assumes that the presence of one or more copies of the risk allele increases disease risk; thus, *DD* or *Dd* would have a higher disease risk than *dd*. Finally, a recessive model assumes that two copies of the risk allele are needed to alter the risk of disease, so individuals possessing *DD* genotypes would be compared to those with *Dd* or *dd* genotypes (Bush and Moore, 2012).

Pseudo–heritability was estimated for each model as:

$$h^{2}=\frac{\sigma_{g}^{2}}{\left(\sigma_{g}^{2}+\sigma_{e}^{2}\right)}$$

where, $\sigma_{g}^{2}$ represented the estimated genetic variance, and $\sigma_{e}^{2}$ represented the estimated random residual (error) variance (Kang et al., 2010). Test statistics can be artificially inflated due to underlying population structure resulting in spurious associations; therefore, genomic inflation factors were assessed for each model using the “Calculate an Approximate Lambda from a Set of P-Values” script in SVS (version 8.9.0; Golden Helix, Bozeman, Montana, United States). The genomic inflation factor was expressed as:

$$\lambda =\frac{\text{Median}\;\chi^{2}}{\text{Expected}\;\text{Median}\;\chi^{2}}$$

where, $\chi^{2}$ was the chi–square values and λ < 1.01, λ < 1.05, or λ > 1.1 suggest minimally, moderately, or highly inflated test statistics, respectively (Aulchenko, 2015). For this study, SNP were moderately associated with PRV infection status when the *P* value was between 5 × 10^−7^ and 1 × 10^−5^ and strongly associated when the *P* value was <5 × 10^−7^ (Wellcome Trust Case Control Consortium, 2007).

### 2.9 Gene set enrichment analysis—single nucleotide polymorphism

To investigate gene sets associated with PRV infection status, we performed GSEA–SNP using the GenGen software package (Wang et al., 2007). The GenGen algorithm was adapted from GSEA (Subramanian et al., 2005) for specific application to GWAS data. Briefly, SNP are mapped to genes and the test statistic [*P* value or chi–square value ( $\chi^{2}$ )] of the most significant SNP mapped to the gene is used as a proxy for gene significance. Genes are then ranked based on their significance (e.g., largest $\chi^{2}$ test statistic to smallest) and an enrichment score (ES), a weighted Kolmogorov–Smirnov–like running sum statistic that reflects the degree to which the genes in a gene set are overrepresented at the top of the ranked gene list, is then calculated for each gene set. The ES of a gene set will be higher if its genes are at the top of the ranked list.

Larger genes may be ranked higher than smaller genes due to increased numbers of SNP within the gene, thus artificially inflating pathway significance (Holmans et al., 2009; Wang et al., 2010; Fridley and Biernacka, 2011). The GenGen algorithm accounts for variable gene size and linkage disequilibrium among SNP in a gene using a two-step approach (Wang et al., 2007; Wang et al., 2009; Wang et al., 2011). First, phenotype–based permutations are used to describe the distribution of the test statistics under the null hypothesis that there is no association between genotype and phenotype. For each permutation, the ES is calculated as described above. Second, the ES derived from the empirical data and the permutations are used to compute normalized enrichment scores (NES). The nominal *P* value of each ES is calculated from the permutations and a false discovery rate (FDR) is used to account for multiple–hypothesis testing.

To obtain input files for the GenGen algorithm, we reran the GWAS for each of the three study populations using a GRAMMAR (Genome Wide Rapid Association using Mixed Model and Regression; Aulchenko et al., 2007a) approach with the GenABEL package (Aulchenko et al., 2007b) in R (version 4.1.1; R Core Team, 2021). First, an additive polygenic model was fit with a genomic kinship matrix [identity–by–state (IBS)] and binomial distribution to derive environmental residuals. Note that for each study population, we used the same fixed effects in the polygenic model as we used for the EMMAX implemented in SVS. Second, environmental residuals were used as the trait in the “qtscore” function in GenABEL, which performs a score test for association between SNP and a trait of interest. The resulting *P* values were converted to chi–square values using the “qchisq” function in the R stats package (version 4.1.1; R Core Team, 2021). We performed 10,000 phenotype–based permutations (without replacement) on the environmental residuals using the “sample” function in R (version 4.1.1; R Core Team, 2021) and for each permutation the score test was performed, and chi–square values were generated.

Using the Sscrofa11.1 genome assembly (29,862 genes; NCBI *Sus scrofa* Annotation Release 106; Assembly accession GCF_000003025.6; National Center for Biotechnology Information (NCBI), 1988; Warr et al., 2020), the 56,024 SNP from the GWAS were mapped to genes if they fell within the gene or within a haplotype block of the gene (74 kb; 37 kb 5′ and 37 kb 3′). The average haplotype block size was calculated using the comprehensive study population (*n* = 2,490) and the default parameters in SVS (version 8.9.0; Golden Helix, Bozeman, Montana, United States) which implements the methods previously described by Gabriel et al. (2002). Human (*Homo sapiens*) gene sets are highly curated, whereas less information is available for swine; therefore, we referenced human gene sets (*n* = 12,121) available from five databases: BioCarta (*n* = 289; Nishimura, 2001), Gene Ontology (GO; *n* = 9,996; Ashburner et al., 2000; The Gene Ontology Consortium et al., 2023), Kyoto Encyclopedia of Genes and Genome (KEGG; *n* = 186; Kanehisa and Goto, 2000), Protein Analysis THrough Evolutionary Relationships (PANTHER; *n* = 151; Mi and Thomas, 2009; Thomas et al., 2022), and Reactome (*n* = 1,499; Gillespie et al., 2021; Supplementary Table S1). Gene sets were filtered for redundancy to increase our power in respect to false discovery rate (see Supplementary Material; Subramanian et al., 2005; Wang et al., 2007; Broad Institute, 2023). The minimum gene set size (i.e., the number of genes in a gene set) was set to 15 and the maximum gene set size was set to 500. As recommended by Wang et al. (2007), the “largest_es” argument was implemented so that only the largest enrichment scores were used instead of enrichment scores with the largest absolute value. When evaluating gene expression data, negative enrichment scores may be attributed to negative regulation making absolute values appropriate whereas this is not the case with GWAS data.

The established threshold for statistically significant gene sets is FDR <0.25 (Subramanian et al., 2005; Wang et al., 2009; Wang et al., 2011; Broad Institute, 2023). However, in the absence of statistically significant gene sets, evaluating the top ranked gene sets is recommended as these gene sets may yet be biologically meaningful for PRV infection (Broad Institute, 2023). For the current study, gene sets were considered top ranked (i.e., putatively associated with PRV infection status) if their NES fell within the top 0.1% percentile (NES >0.999 quantile), aligning with thresholds implemented by Lee et al. (2013), Del Corvo et al. (2017), Kiser et al. (2018), and Neupane et al. (2018). Genes that positively contributed to the ES within a gene set, termed leading edge genes (LEGs), were also identified during the analyses.

## 3 Results

### 3.1 Study populations

A detailed description of each study population is provided in Table 1. Briefly, the comprehensive study population consisted of 2,490 feral swine (1,062 cases and 1,428 controls) sampled across 18 states, primarily in the southeastern US, and six bioclimatic regions. The HTS study population was comprised of approximately half of the samples in the comprehensive study population, with 1,183 feral swine (653 cases and 530 controls) sampled from six bioclimatic regions across eight states. The smallest study population was CSHTS which consisted of 291 feral swine (164 cases and 127 controls). Given the specified bounds on temporal variation, the CSHTS study population only included feral swine sampled from Florida, South Carolina, and Texas. Within these three states, five unique bioclimatic regions were represented.

**TABLE 1:** Descriptions of three complementary study populations used to identify genomic regions associated with pseudorabies virus (PRV) infection status in naturally infected feral swine (*Sus scrofa*) within the contiguous United States.

		Phenotype classification^c^		Sex		PRV true seroprevalence^d^			Percent European wild boar ancestry^e^		
Population^a^	Watershed-year combinations^b^	Case	Control	Male	Female	Min	Median	Max	Min	Median	Max
Comprehensive	291	1,062	1,428	1,075	1,415	2.60	35.8	88.5	0.80	27.0	79.2
HTS	126	653	530	527	656	11.0	54.2	88.5	2.90	20.1	69.2
CSHTS	27	164	127	126	165	24.2	54.4	88.5	4.90	24.9	69.2

^a^ Study population examined: a comprehensive population consisting of adult feral swine throughout the invaded range within the contiguous United States (Comprehensive), a population of adult feral swine experiencing high PRV infection pressure with high temporal variability (HTS), and a population of adult feral swine experiencing temporally stable high PRV infection pressure (CSHTS).

^b^ The number of unique watershed–year combinations represented in the study population.

^c^ The number of pseudorabies antibody positive swine (case) and pseudorabies antibody negative swine (control) in the study population.

^d^ True seroprevalence of PRV predicted using hierarchical Bayesian modeling.

^e^ Percent European wild boar ancestry calculated using the methods of Smyser et al. (2020).

### 3.2 Model selection

For each of the three study populations, we identified multiple competing models within two AIC units of the best model (ΔAIC ≤2; Supplementary Tables S2–S4); therefore, cumulative covariate weights were calculated to evaluate the relative importance of each predictor (Doherty et al., 2012). Based on these weights, PRV true seroprevalence (0.83) was considered informative for predicting PRV infection status within the comprehensive study population. Given that the true seroprevalence for PRV was used to define the HTS and CSHTS study populations, true seroprevalence and interactions that included true seroprevalence were not evaluated as potential predictors for these models. Percent European wild boar ancestry was informative for predicting PRV infection status within the HTS and CSHTS populations with cumulative covariate weights of 0.82 and 0.72, respectively. Quantile–quantile (Q–Q) plots (Supplementary Figure S1), as well as genomic inflation factors (Supplementary Table S5), demonstrated that underlying population stratification was adequately corrected for in every model and test statistics would not be artificially inflated.

### 3.3 Pseudo–heritability estimates and candidate genes

**FIGURE 1:**
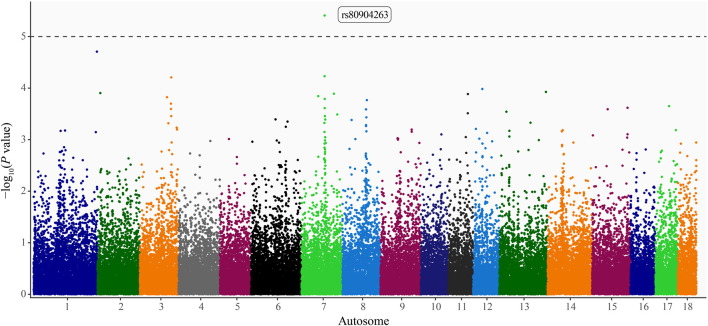
Manhattan plot of loci associated with pseudorabies virus (PRV) infection status in naturally infected feral swine (*Sus scrofa*) within the contiguous United States. Autosome position (Sscrofa 11.1 genome assembly) on the x-axis and -log_10_(*P* values) on the y-axis. Significance threshold denoted by the dashed line (*P* < 1 × 10^−5^; Wellcome Trust Case Control Consortium, 2007). A dbSNP Reference SNP (rs) number is provided for the significant locus (Sherry et al., 2001).

Pseudo–heritability estimates using the comprehensive study population were low (0.10 ± 0.03–0.13 ± 0.04), whereas estimates calculated using the HTS population were moderate (0.30 ± 0.06–0.38 ± 0.08). The pseudo–heritability estimates from the CSHTS population were low (0.13) with high standard errors (±0.13–0.16). Using GWAS, no significant SNP were identified for the Comprehensive or HTS study populations. Moreover, no significant loci were identified using the dominance or recessive models. An intronic variant within A–Kinase Anchoring Protein 6 (*AKAP6*) gene, rs80904263 (base pair position 7:67,174,060), was moderately associated with PRV infection status in the additive model using the CSHTS study population (*P* value = 3.90 × 10^−6^; Figure 1).

### 3.4 Enriched gene sets

Using the 74 kb haplotype block, 44,744 SNP from the GWAS were successfully mapped to 22,810 genes from the Sscrofa11.1 genome assembly. Of the 12,121 human gene sets, 5,843 were retained for gene–set enrichment analysis based on redundancy filtering and the minimum and maximum size criteria. Seventeen top ranked gene sets (Table 2) were identified using the 0.1% percentile of the NES distribution for each study population (Supplementary Figure S2). Leading edge genes for each gene set are provided in Supplementary Table S6.

**TABLE 2:** Gene sets associated with pseudorabies virus (PRV) infection status in adult feral (*Sus scrofa*) sampled across the invaded range in the United States using gene set enrichment analysis of single nucleotide polymorphism data (GSEA–SNP).

Population^a^	Gene set^b^	NES^c^	*P* value	FDR^d^	Genes^e^	LEGs^f^
Comprehensive	Muscle cell cellular homeostasis (GO:0046716)	3.50	0.0001	1.00	17	4
Comprehensive	Negative regulation of cellular protein localization (GO:1903828)	3.31	0.0004	1.00	93	36
Comprehensive	Spinal cord motor neuron differentiation (GO:0021522)	3.19	0.0008	1.00	30	6
Comprehensive	Ventral spinal cord development (GO:0021517)	3.03	0.0007	1.00	42	8
Comprehensive	Protein localization to cilium (GO:0061512)	2.98	0.0020	1.00	40	15
Comprehensive	Epithelial cell apoptotic process (GO:1904019)	2.92	0.0021	1.00	90	43
HTS	Membrane depolarization during cardiac muscle cell action potential (GO:0086012)	3.09	0.0005	1.00	21	8
HTS	Cellular response to sterol (GO:0036315)	3.08	0.0005	1.00	22	11
HTS	Sumoylation of transcription factors (Reactome)	3.06	0.0008	1.00	15	7
HTS	Cell adhesion mediator activity (GO:0098631)	3.02	0.0011	1.00	54	27
HTS	Negative regulation of synapse organization (GO:1905809)	2.90	0.0017	1.00	21	11
CSHTS	Glycosaminoglycan biosynthesis chondroitin sulfate (KEGG)	3.21	0.0004	1.00	20	11
CSHTS	Regulation of calcium ion transport into cytosol (GO:0010524)	3.06	0.0011	1.00	86	26
CSHTS	Actin filament organization (GO:0007015)	3.05	0.0020	1.00	336	113
CSHTS	Pyruvate metabolism and citric acid (TCA) cycle (Reactome)	3.03	0.0011	1.00	44	23
CSHTS	Thioester metabolic process (GO:0035383)	2.90	0.0027	1.00	35	17
CSHTS	Sphingolipid metabolic process (GO:0006665)	2.88	0.0023	1.00	64	21

^a^ Study population examined: a comprehensive population consisting of adult feral swine throughout the invaded range within the contiguous United States (Comprehensive), a population of adult feral swine experiencing high PRV infection pressure with high temporal variability (HTS), and a population of adult feral swine experiencing temporally stable high PRV infection pressure (CSHTS).

^b^ Name of gene set and database: Gene Ontology (GO), Kyoto Encyclopedia of Genes and Genome (KEGG), Reactome.

^c^ Normalized enrichment score.

^d^ False discovery rate.

^e^ Number of genes in the gene set.

^f^ Number of leading edge genes in the gene set.

Of the top ranked gene sets (the top 0.1% based on NES), muscle cell cellular homeostasis and negative regulation of cellular protein localization had significant *P* values (*P* < 0.01), but high FDR (FDR = 1.00). However, the FDR for these gene sets fell to 0.458 and 0.486 respectively when a more conservative pruning methodology was implemented (Jaccard similarity coefficient >0.5) and approximately half the number of gene sets were removed (see Supplementary Material).

## 4 Discussion

Opportunistic serological data, amassed under USDA disease surveillance efforts, provided a unique opportunity to enhance our understanding of PRV epidemiology and disease dynamics by coupling this data with host genetics. Results from the GWAS suggest that variation in PRV infection status, as determined by the presence/absence of antibodies, may, in part, be attributed to genetics. Further, metabolic pathways that may be biologically relevant for PRV infection in free–ranging feral swine were identified through complimentary, pathway–based analyses of the GWAS data.

Pseudo–heritability estimates were low to moderate across our three study populations, which aligns with previous heritability estimates for disease presence/absence in domestic swine (Henryon et al., 2001; Henryon et al., 2003; Bishop and Woolliams, 2010). We identified one intronic SNP within *AKAP6* on autosome 7 that was moderately associated with PRV infection status. In swine, the highest expression levels of *AKAP6* are in the heart and skeletal muscle (Li et al., 2017). Gross and histologic lesions are not typically described in the heart muscle of swine infected with PRV (Yang et al., 2016); however there have been reports of domestic dogs (*Canis lupus familiaris*) inoculated with PRV that developed heart lesions with herpes-like viral particles observed by electron microscopy in autonomic ganglia and myocardial endothelial cells (Olson and Miller, 1986).

Previous research on susceptibility/resistance to PRV in domestic swine identified quantitative trait loci (QTL) on autosomes 5, 6, 9, and 13 that were associated with the appearance/non–appearance of neurologic symptoms following PRV challenge. In addition, QTL associated with rectal temperature were identified on autosomes 2, 4, 8, 10, 11, and 16 (Reiner et al., 2002). Similarly, a gene expression analysis using brain and lung tissues of naturally infected commercial breed piglets identified differentially expressed genes on autosomes 8, 9, and 13 (Yuan et al., 2009). In studying susceptibility/resistance in free–ranging, naturally infected European wild boar, (Fabbri et al., 2021) identified intergenic and intronic SNP on autosomes 12 and 18, respectively. Given that previous studies focused on different lineages under unique selection pressure it is reasonable that the findings differed from ours (Bruns and Stalder, 2019).

Based on previous work with swine brucellosis in feral swine, it was hypothesized that enriched gene sets would include processes involved in the immune response or, more specifically, the humoral response (Pierce et al., 2020); however, results from our study did not find any significant associations with genes sets involved in the host immune response to infection. Instead, gene sets identified in this study were associated with neuronal development, muscle cells, calcium transport, and actin filaments, protein localization, and cellular metabolic processes (particularly lipid metabolic processes). Previous PRV research in both *in vivo* and *in vitro* models demonstrated similar findings.

Three of the top ranked gene sets in our study were related to neuronal development including negative regulation of synapse organization, ventral spinal cord development, and spinal cord motor neuron differentiation. Similarly, Yuan et al. (2009) demonstrated that genes associated with nervous system development and synaptic transmission were upregulated in brain and lung tissue while investigating the transcriptional response of commercial breed piglets to natural PRV infection. Pseudorabies virus infections often occur with infection of neurons in the periphery and then spread to the central nervous system (CNS) via synapses linking neurons. Proteomic analysis revealed that synapse organization proteins were altered in mice (*Mus musculus*) infected with Bartha K61-an attenuated strain of PRV. Moreover, many of these proteins showed a decreasing tendency, suggesting that PRV infection could inhibit host synaptic transmission (Zeng et al., 2018).

In young swine, PRV infection can be characterized by neuronal clinical signs, including ataxia, incoordination, convulsions, and paralysis. Histologically, nonsuppurative inflammation has been observed in the brain, spinal cord, and ganglia as characterized by neuronal degeneration and necrosis, neuronophagia, and microglial activation (Sehl and Teifke, 2020). Neurons clearly play a large role in PRV infection in swine, but the association between neuronal development and PRV infection status of swine remains unknown.

Gene sets associated with muscle cells, calcium transport, and actin filaments were also enriched in our study, specifically muscle cell cellular homeostasis, membrane depolarization during cardiac muscle cell action potential, regulation of calcium ion transport into cytosol, and actin filament organization. Although PRV does not demonstrate a tropism for muscle cells in swine, these cells could be indirectly affected by the systemic inflammatory response elicited by the virus as well as other stresses on muscle cells induced by infection (i.e., muscle tremors, convulsions). Systemic inflammation, evident by PRV–associated pyrexia and anorexia, puts animals in a state of negative energy balance as immune responses are energetically expensive and often accompanied by decreased feed intake. Currently, there is not a clear connection between muscle cells and PRV infection status in swine. However, as mentioned previously, domestic dogs inoculated with PRV have demonstrated varying degrees of heart lesions ranging from myocardial degeneration to myolysis (Olson and Miller, 1986). Moreover, upregulated pig gene homologues have been identified in the calcium signaling pathway in response to PRV infection in commercial breed piglets (Yuan et al., 2009).

Actin filaments are present in most cells providing shape and mechanical properties to the cytoplasm but are well known for their abundance in muscle cells. Herpesviruses such as PRV have been reported to utilize the cytoskeleton and modify the actin filaments of host cells for efficient viral entry and replication in the nucleus (Lyman and Enquist, 2009). Specifically, US3 a tegument protein of PRV has been shown to lead to actin filament disruption in order to facilitate viral transport to the nucleus and bind to antiviral proteins in the host cell (Jacob et al., 2015; Zhang et al., 2023). How efficiently a virus gains host entry, replicates, and exits the host cell, can impact its ability to establish infections and even avoid immune detection. Targeting the actin filament organization of its host cells could impact PRV infection status. Pig gene homologues involved in the regulation of actin cytoskeleton were upregulated in piglets naturally infected with PRV infection (Yuan et al., 2009).

Two of the enriched gene sets (i.e., protein localization to cilium and negative cellular protein localization) were involved protein localization. The association between PRV infection and cilium protein transport may result from genetic variabilities among hosts, resulting in varying degrees of cilium function and/or cilium sensory capabilities. Since the upper respiratory tract is the initial site of PRV invasion and replication, PRV has evolved effective mechanisms to overcome the mucosal epithelial barrier, which is coated in a mucus layer, and together, serve as the primary defense against infection (Wittmann et al., 1980). The association noted here may be indicative of small variations in cilium function that allow PRV to more readily evade the mucosal barrier and allow for PRV invasion and establishment of infection.

Negative cellular protein localization refers to regulatory pathways that prevent normal localization of proteins to specific sites within the cell. All aspects of the life cycle of PRV—infection, latency and replication—are dependent on host cell machinery and protein transport mechanisms (Pomeranz et al., 2005). Therefore, it is not surprising to observe this type of association between regulation of host cell protein transport and PRV infection.

Four gene sets related to cellular metabolic processes were associated with PRV infection status including cellular response to sterol, sphingolipid metabolism, thioester metabolic process, and pyruvate metabolism and citric acid (TCA) cycle. Cell entry of PRV has been shown to be inhibited by the depletion of sphingomyelin, which is an abundant sphingolipid in cell membranes (Pastenkos et al., 2019). In addition, the depletion of cholesterol in the cell membrane also impaired the infectivity of different PRV strains (Ren et al., 2011). In a study analyzing differentially expressed metabolites in porcine kidney (PK) cells infected with PRV compared to uninfected cells, 35% belonged to lipid metabolites (Liu et al., 2022). Research with PRV infected porcine alveolar macrophages had similar results, with over 50% of altered metabolites being lipid and lipid–like molecules (Yao et al., 2021). Thus, these data would support differential sphingolipid metabolism and generally lipid metabolism as serving a role in the susceptibility of swine to PRV infection. However, metabolic networks studied using mass spectrometry after PK cell infection with PRV showed the tricarboxylic acid (TCA) cycle had little effect on viral replication (Gou et al., 2021).

Gene set enrichment analysis conducted using high density genotypes of naturally infected European wild boar classified metabolic process as an enriched gene set (Fabbri et al., 2021). *In vitro* work with various cell lines infected with PRV found that many altered metabolites were lipids or lipid–like molecules (Yao et al., 2021; Liu et al., 2022). Given the pressures and stressors that affect overall host fitness, lipid metabolic activity could be biologically meaningful for PRV infection. Infections can affect host cells directly or indirectly. For example, PRV, as an enveloped virus, alters the cellular membranes during replication and release from the cell, which could explain changes in lipid metabolic pathways. Metabolic activity of cells has been shown to be a factor driving permissibility to viral replication (Munger et al., 2008), and through metabolomic approaches, various studies have demonstrated viral–driven alterations in metabolic pathways of host cells (Plaza et al., 2016). Thus, the potential role of metabolism in host susceptibility plausible. Nevertheless, given the complexity of metabolic pathways, it is difficult to determine at this time which specific component may be involved in resistance/susceptibility to PRV infection.

Utilizing retrospective field data enabled large sample sizes and increased statistical power to detect genomic regions associated with PRV infection status; however, these data were limited in that only binary disease phenotypes based on serological data were collected. The humoral immune response to PRV is stable, and it is thought to last throughout the lifetime of the individual; therefore, it is difficult to use serological data alone to infer timing of infection (Mettenleiter et al., 2019). Moreover, interpreting the associations between the identified genomic regions and PRV infection status is challenging because seropositivity is an indication of infection or exposure, but does not predict susceptibility to disease. Similarly, a seronegative animal may be indicative of a resistant animal or incomplete exposure to PRV.

Future studies would benefit from continuous phenotypes that allow individuals to be characterized as resistant, tolerant, or susceptible (Bishop and Woolliams, 2014). For example, viral shedding could be used alongside serology to further characterize infection status. Previous work has demonstrated that serology alone may underestimate the true prevalence of PRV infection in feral swine and that viral detection via PCR could identify infected yet seronegative animals (Hernández et al., 2018b). Although this is easier to accomplish under experimental settings where variables such as the timing of infection are known, clinical signs can be tracked, and animals can be sampled longitudinally, it may be feasible to accomplish this in free–ranging populations.

Continuing to improve our understanding of the epidemiology and disease dynamics of PRV in feral swine will be important for refining disease surveillance methods and assessing the ongoing risk of spillover into commercial swine populations. Feral swine control resources are limited, and priorities are dynamic. Data generated from this study and surveillance programs can help guide control efforts to prioritize regions with the greatest PRV prevalence. Overall, if PRV can be reduced in feral swine populations, the risk of spillover into commercial swine herds is lowered, thus helping to secure the health of US pork production.

## Data Availability

The original data presented in the study are deposited in Dryad (https://doi.org/10.5061/dryad.zgmsbccjh). Information for existing publicly accessible data can be found in the article/Supplementary Material.
