# Supplementary material for: Genomic regions associated with pseudorabies virus infection status in naturally infected feral swine (Sus scrofa)
Source: Front Genet. 2023 Nov 23;14:1292671. doi: 10.3389/fgene.2023.1292671 (PMC10701407; doi:10.3389/fgene.2023.1292671)
Supplement: Supplementary file 1 [file Table1.docx]

Supplementary Material

# Watershed level prediction of true seroprevalence

Recent studies have identified a need to include detection error (i.e., sensitivity or probability that a disease is detected when it is present, and specificity or the probability that a disease is not detected when it is absent) in ecological models using disease prevalence (Tabak et al 2019, Lachish et al, 2012; McClintock et al., 2010; Royle & Link, 2006). Several studies have demonstrated that assuming testing is perfect when the sensitivity is less than perfect can result in underestimates of pathogen prevalence (Miller 2017; DiRenzo et al., 2018; Lachish et al., 2012; Miller et al., 2012) and that ignoring sensitivity and specificity errors may lead to erroneous conclusions about the risk associated with pathogen transmission (Tabak et al. 2019). Studies have also demonstrated that imperfect detection can be related to host infection intensity resulting in nonrandom bias in pathogen detection (DiRenzo et al., 2018; Lachish et al., 2012).

We constructed an occupancy model using a hierarchical state-space formulation (Royle and Kéry, 2007) for modeling individual infection probability and for predicting watershed true seroprevalence. We express the model by its two component processes that are the observations, $y_{ij}$, conditional on the unobserved state process (i.e.,$y_{ij}|Z_{j}$) and, the unobserved or partially observed state process, $Z_{j}$,where *i* indexes the cohort of pigs sampled for disease and *j* indexes the watershed that they were located. The observation model specified conditional on the latent process, $\boldsymbol{Z}_{\boldsymbol{j}}$, is given by:

| $y_{ij}\vert Z_{j} \sim Binomial\left( Z_{j}\rho+\left( 1-Z_{j} \right)\left( 1-\phi\right),n_{ij} \right)$ | Eq. 1 |
| --- | --- |

where $\rho$ is the true positive detection probability (i.e., sensitivity) and $\left( 1-\phi\right)$ is the false positive detection probability (i.e., specificity) across all watersheds and $n_{ij}$ is the size of the cohort. This formulation, which is common in epidemiology (McClintock et al., 2010; Christensen et al., 2011) and increasingly common in species occupancy models (Royle and Link, 2006; Miller et al., 2011), provides a prediction of the true state, in our case the probability of being infected or not infected, accounting for false positive and false negative errors allowing for population level true seroprevalence to be predicted.

The true detection probability $\rho$, and false detection probability, $\phi$, can result from a diversity of processes including diagnostic test error (Branscum et al., 2005), population immunity (Pepin et al., 2017), and differences in detectability of the pathogen given infection status of the animal (Jennelle et al., 2007). The diagnostic tests used for pseudorabies were developed and validated for domestic animals but performance of diagnostic tests are often different for wildlife (Stallknecht, 2007). The beta distribution was used to account for true and false detection error arising from diagnostic error and other processes. We used a parameterization common in epidemiological studies (Branscum et al., 2005; Christensen et al., 2011) that is often termed the expert beta. We parameterized $\rho$ and $\phi$ with respect to the mean for the diagnostic error using,

| $\rho\sim\mathrm{Beta}\left( \mu_{\rho} \sigma_{\rho}, \left( 1-\mu_{\rho} \right)\sigma_{\rho} \right)$ | Eq. 2 |
| --- | --- |
| $\phi\sim\mathrm{Beta}\left( \mu_{\phi} \sigma_{\phi}, \left( 1-\mu_{\phi} \right)\sigma_{\phi} \right)$ | Eq. 3 |

where $\mu_{\rho}$ and $\mu_{\phi}$ are beta distributed hyper-priors for the detection mean and $\sigma_{\rho}$ and $\sigma_{\phi}$ are gamma distributed hyper-priors for the detection variance. This parameterization allows the mean and variance for each detection probability to be defined in terms of the confidence in the available data (Christensen et al., 2011). We assumed in the absence of other contributors to observation error that the true and false detection rates would approach those of the diagnostic test. Deviations in the posterior predictive distribution from the diagnostic test values would indicate other contributions to detection errors are present. For this purpose, we assumed $\mu_{\rho}$ and $\mu_{\phi}$ were, with 65% confidence, to be the reported diagnostic test error rate, and we assumed with 95% confidence that the true values of $\mu_{\rho}$ and $\mu_{\phi}$ were greater than 0.6. This parameterization allowed some uncertainty in the true value of $\mu_{\rho}$ and $\mu_{\phi}$ with the null hypothesis that the mean value is the same as the diagnostic test.

Posterior distributions of the latent states and parameters of interest were predicted using Markov chain Monte Carlo (MCMC) methods using three chains with diffuse initial conditions (Brooks and Gelman, 1998). Each parameter and latent state was predicted by sampling from the relevant posterior distributions using Gibbs sampling implemented in JAGS (Plummer, 2014) and the runjags package (Denwood, 2016) in the R computing environment (R Core Team, 2021). The MCMC procedure was run until convergence of the distributions of samples of all model parameters was achieved. Once convergence was assured posterior inference was based on 20,000 samples from the MCMC chains. Convergence was evaluated by visual inspection of trace plots, the Gelman-Rubin diagnostic (Gelman and Rubin, 1992), and the Heidelberg-Welch diagnostic (Heidelberger and Welch, 1983). Convergence diagnostics and statistical analysis of the model output was done using the R package coda (Plummer et al., 2006).

**References**

Branscum, A., Gardner, I., and Johnson, W. (2005). Estimation of diagnostic-test sensitivity and specificity through Bayesian modeling. *Preventive veterinary medicine* 68(2)**,** 145-163.

Brooks, S.P., and Gelman, A. (1998). General methods for monitoring convergence of iterative simulations. *Journal of computational and graphical statistics* 7(4)**,** 434-455.

Christensen, R., Johnson, W., Branscum, A., and Hanson, T.E. (2011). *Bayesian ideas and data analysis: an introduction for scientists and statisticians.* CRC Press.

Denwood, M.J. (2016). runjags: An R package providing interface utilities, model templates, parallel computing methods and additional distributions for MCMC models in JAGS. *Journal of Statistical Software* 71(9)**,** 1-25.

Gelman, A., and Rubin, D.B. (1992). Inference from iterative simulation using multiple sequences. *Statistical science***,** 457-472.

Heidelberger, P., and Welch, P.D. (1983). Simulation run length control in the presence of an initial transient. *Operations Research* 31(6)**,** 1109-1144.

Jennelle, C.S., Cooch, E.G., Conroy, M.J., and Senar, J.C. (2007). State-specific detection probabilities and disease prevalence. *Ecological Applications* 17(1)**,** 154-167.

McClintock, B.T., Nichols, J.D., Bailey, L.L., MacKenzie, D.I., Kendall, W., and Franklin, A.B. (2010). Seeking a second opinion: uncertainty in disease ecology. *Ecology letters* 13(6)**,** 659-674.

Miller, D.A., Nichols, J.D., McClintock, B.T., Grant, E.H.C., Bailey, L.L., and Weir, L.A. (2011). Improving occupancy estimation when two types of observational error occur: non‐detection and species misidentification. *Ecology* 92(7)**,** 1422-1428.

Pepin, K.M., Kay, S.L., Golas, B.D., Shriner, S.S., Gilbert, A.T., Miller, R.S., et al. (2017). Inferring infection hazard in wildlife populations by linking data across individual and population scales. *Ecology Letters* 20(3)**,** 275-292.

Plummer, M. (2014). JAGS: A program for analysis of Bayesian graphical models using Gibbs sampling, 2003. *URL* [*http://sourceforge.net/projects/mcmc-jags*](http://sourceforge.net/projects/mcmc-jags).

Plummer, M., Best, N., Cowles, K., and Vines, K. (2006). CODA: convergence diagnosis and output analysis for MCMC. *R news* 6(1)**,** 7-11.

R Core Team (2021). *R: A language and environment for statistical computing* [Online]. Vienna, Austria: R Foundation for Statistical Computing. Available: <https://www.R-project.org/> [Accessed 2023].

Royle, J.A., and Kéry, M. (2007). A Bayesian state‐space formulation of dynamic occupancy models. *Ecology* 88(7)**,** 1813-1823.

Royle, J.A., and Link, W.A. (2006). Generalized site occupancy models allowing for false positive and false negative errors. *Ecology* 87(4)**,** 835-841.

Stallknecht, D. (2007). "Impediments to wildlife disease surveillance, research, and diagnostics," in *Wildlife and Emerging Zoonotic Diseases: The Biology, Circumstances and Consequences of Cross-Species Transmission,* eds. J.E. Childs, J.S. Mackenzie & J.A. Richt. (Berlin, Heidelberg: Springer), 445-461.

# Gene set redundancy filtering

We referenced human (*Homo sapiens*) gene sets (n = 12,121) from five databases: BioCarta (Nishimura, 2001), Gene Ontology (GO; Ashburner et al., 2000; The Gene Ontology Consortium et al., 2023), Kyoto Encyclopedia of Genes and Genome (KEGG; Kanehisa and Goto, 2000), Protein Analysis THrough Evolutionary Relationships (PANTHER; Mi and Thomas, 2009; Thomas et al., 2022), and Reactome (Gillespie et al., 2021). Gene sets derived from BioCarta, Gene Ontology, KEGG, and Reactome were obtained from the Molecular Signatures Database (MsigDB; Subramanian et al., 2005; Liberzon et al., 2011; Table S1). Gene sets derived from PANTHER were obtained from PANTHER Pathway (Table S1; Mi and Thomas, 2009; Thomas et al., 2022).

**Table S1.** Descriptions of human (*Homo sapiens*) gene sets referenced for gene set enrichment analysis of single nucleotide polymorphism data (GSEA-SNP).

| **Database**^1^ | **Description** | ***n***^2^ | **Source**^3^ | **Access Date** |
| --- | --- | --- | --- | --- |
| BioCarta | Canonical Pathways gene sets | 289 | <http://www.gsea-msigdb.org/gsea/msigdb/> | 14-Feb-20 |
| GO | GO Biological Process ontology gene sets  GO Cellular Components ontology gene sets  GO Molecular Function ontology gene sets | 9,996 | <http://www.gsea-msigdb.org/gsea/msigdb/> | 14-Feb-20 |
| KEGG | Canonical Pathways gene sets | 186 | <http://www.gsea-msigdb.org/gsea/msigdb/> | 14-Feb-20 |
| Reactome | Canonical Pathways gene sets | 1,499 | <http://www.gsea-msigdb.org/gsea/msigdb/> | 14-Feb-20 |
| PANTHER |  | 151 | <https://www.pantherdb.org/pathway/> | 14-Feb-20 |

^1^ Database from which the gene sets were derived. Gene Ontology (GO), Kyoto Encyclopedia of Genes and Genome (KEGG), Protein Analysis THrough Evolutionary Relationships (PANTHER).

^2^ Number of gene sets obtained from each database.

^3^ Molecular Signatures Database (<http://www.gsea-msigdb.org/gsea/msigdb/>), PANTHER Pathway (<https://www.pantherdb.org/pathway/>).

To reduce redundancy between gene sets, we filtered highly similar gene sets using Jaccard similarity coefficients and hierarchical clustering (Broad Institute, 2020; 2021). First, we computed Jaccard similarity coefficients for every pairwise combination of gene sets as:

$$J(A,B)=\frac{|A\bigcap B|}{|A\bigcup B|}$$

where, $A$represented gene set 1, and $B$ represented gene set 2. Note that for the Jaccard similarity coefficient calculation we only retained genes within a gene set if any one of the SNP from the GWAS input fell within the gene or within a haplotype block of the gene (74 kb). Gene set pairs with a Jaccard similarity coefficient >0.85 were considered highly similar (n = 672 individual gene sets) and were used to generate a dissimilarity matrix $[1- J\left( A,B \right)$]. The “hclust” function with default parameters in the R package stats (version 4.1.1; R Core Team, 2021) was used to perform hierarchical cluster analysis on the dissimilarity matrix. The “cutree” function in the R package stats (version 4.1.1; R Core Team, 2021) was used to cut the dendrogram at a height of 0.90 resulting in 235 clusters (Memon et al., 2021). Gene sets assigned to the same cluster were then filtered using an iterative approach in which the most basal gene set (i.e., greatest leaf height) was retained. In the instance that two gene sets had the same leaf height, the gene set with the largest number of genes was retained. If both leaf height and gene set size were the same, then we retained the first gene set listed in the cluster. After filtering for redundancy, 11,684 gene sets were retained for gene-set enrichment analysis. To determine the effect of reducing the number of gene sets on the false discovery rate (FDR) for the gene set enrichment analysis, we implemented a more conservative prune using a Jaccard similarity coefficient >0.5, resulting in 6,407 gene sets.

**References**

Ashburner, M., Ball, C.A., Blake, J.A., Botstein, D., Butler, H., Cherry, J.M., et al. (2000). Gene Ontology: tool for the unification of biology. *Nature Genetics* 25(1)**,** 25-29. doi: 10.1038/75556.

Broad Institute (2020). *MSigDB v7.0 Release Notes* [Online]. Available: <https://software.broadinstitute.org/cancer/software/gsea/wiki/index.php/MSigDB_v7.0_Release_Notes> [Accessed 2023].

Broad Institute (2021). *MSigDB v7.3 (Mar 2021)* [Online]. Available: <https://docs.gsea-msigdb.org/#MSigDB/Release_Notes/MSigDB_7.3/> [Accessed 2023].

Gillespie, M., Jassal, B., Stephan, R., Milacic, M., Rothfels, K., Senff-Ribeiro, A., et al. (2021). The reactome pathway knowledgebase 2022. *Nucleic Acids Research* 50(D1)**,** D687-D692. doi: 10.1093/nar/gkab1028.

Kanehisa, M., and Goto, S. (2000). KEGG: kyoto encyclopedia of genes and genomes. *Nucleic Acids Res* 28(1)**,** 27-30. doi: 10.1093/nar/28.1.27.

Liberzon, A., Subramanian, A., Pinchback, R., Thorvaldsdóttir, H., Tamayo, P., and Mesirov, J.P. (2011). Molecular signatures database (MSigDB) 3.0. *Bioinformatics* 27(12)**,** 1739-1740. doi: 10.1093/bioinformatics/btr260.

Memon, D., Rizvi, H., Fromm, G., Lihm, J., Schoenfeld, A.J., Sauter, J.L., et al. (2021). Clinical and molecular features of acquired resistance to immunotherapy in non-small cell lung cancer. *bioRxiv*. doi: <https://doi.org/10.1101/2021.07.21.452854>.

Mi, H., and Thomas, P. (2009). PANTHER pathway: an ontology-based pathway database coupled with data analysis tools. *Methods Mol Biol* 563**,** 123-140. doi: 10.1007/978-1-60761-175-2_7.

Nishimura, D. (2001). BioCarta. *Biotech Software & Internet Report: The Computer Software Journal for Scient* 2(3)**,** 117-120.

R Core Team (2021). *R: A language and environment for statistical computing* [Online]. Vienna, Austria: R Foundation for Statistical Computing. Available: <https://www.R-project.org/> [Accessed 2023].

Subramanian, A., Tamayo, P., Mootha, V.K., Mukherjee, S., Ebert, B.L., Gillette, M.A., et al. (2005). Gene set enrichment analysis: A knowledge-based approach for interpreting genome-wide expression profiles. *Proceedings of the National Academy of Sciences* 102(43)**,** 15545-15550. doi: doi:10.1073/pnas.0506580102.

The Gene Ontology Consortium, Aleksander, S.A., Balhoff, J., Carbon, S., Cherry, J.M., Drabkin, H.J., et al. (2023). The Gene Ontology knowledgebase in 2023. *Genetics* 224(1). doi: 10.1093/genetics/iyad031.

Thomas, P.D., Ebert, D., Muruganujan, A., Mushayahama, T., Albou, L.-P., and Mi, H. (2022). PANTHER: Making genome-scale phylogenetics accessible to all. *Protein Science* 31(1)**,** 8-22. doi: <https://doi.org/10.1002/pro.4218>.

# Model selection results

**Table S2.** Candidate models within two Δ Akaike Information Criterion (AIC) of the “best” model for predicting pseudorabies virus (PRV) infection status of feral swine in the comprehensive study population.

| **Model**^1^ | **Intercept** | **European Wild Boar**^2^ | **Sex** | **PRV True Seroprevalence**^3^ | **df**^4^ | **logLik**^5^ | **AIC**^6^ | **Delta**^7^ | **Weight**^8^ |
| --- | --- | --- | --- | --- | --- | --- | --- | --- | --- |
| Model 1 | -0.3378 |  |  | 0.9014 | 2 | -1487.79 | 2979.6 | 0 | 0.378 |
| Model 2 | -0.3372 | -0.03385 |  | 0.8921 | 3 | -1487.52 | 2981 | 1.46 | 0.182 |
| Model 3 | -0.3661 |  | + | 0.9026 | 3 | -1487.53 | 2981.1 | 1.46 | 0.182 |

^1^ Rows represent candidate models with different combinations of predictors. When a continuous predictor is included in the model, a coefficient is provided. When a categorical predictor is included in the model, a plus sign (+) is provided. Bioclimatic region and an interaction between percent European wild boar ancestry and PRV true seroprevalence were also evaluated as predictors but were not included in the top models.

^2^ Percent European wild boar ancestry calculated using the methods of Smyser et al. (2020).

^3^ True seroprevalence of PRV predicted using hierarchical Bayesian modeling.

^4^ Degrees of freedom of the model.

^5^ Log-likelihood of the model.

^6^ AIC of the model.

^7^ Δ AIC – difference between the AIC of the model and the AIC of the best (or top) model.

^8^ AIC weight of the model.

**Table S3.** Candidate models within two Δ Akaike Information Criterion (AIC) of the “best” model for predicting pseudorabies virus (PRV) infection status of feral swine in the HTS study population.

| **Model**^1^ | **Intercept**^1^ | **Bioclimatic Region** | **European Wild Boar**^2^ | **Sex** | **df**^3^ | **logLik**^4^ | **AIC**^5^ | **Delta**^6^ | **Weight**^7^ |
| --- | --- | --- | --- | --- | --- | --- | --- | --- | --- |
| Model 1 | 0.2106 |  | -0.2255 |  | 2 | -806.198 | 1616.4 | 0 | 0.403 |
| Model 2 | 0.2461 |  | -0.2265 | + | 3 | -805.973 | 1617.9 | 1.55 | 0.186 |
| Model 3 | 0.2839 | + | -0.1715 |  | 7 | -802.114 | 1618.2 | 1.83 | 0.161 |

^1^ Rows represent candidate models with different combinations of predictors. When a continuous predictor is included in the model, a coefficient is provided. When a categorical predictor is included in the model, a plus sign (+) is provided.

^2^ Percent European wild boar ancestry calculated using the methods of Smyser et al. (2020).

^3^ Degrees of freedom of the model.

^4^ Log-likelihood of the model.

^5^ AIC of the model.

^6^ Δ AIC – difference between the AIC of the model and the AIC of the best (or top) model.

^7^ AIC weight of the model.

**Table S4.** Candidate models within two Δ Akaike Information Criterion (AIC) of the “best” model for predicting pseudorabies virus (PRV) infection status of feral swine in the CSHTS study population.

| **Model**^1^ | **Intercept**^1^ | **Bioclimatic Region** | **European Wild Boar**^2^ | **Sex** | **df**^3^ | **logLik**^4^ | **AIC**^5^ | **Delta**^6^ | **Weight**^7^ |
| --- | --- | --- | --- | --- | --- | --- | --- | --- | --- |
| Model 1 | 0.2619 |  | -0.3457 |  | 2 | -195.146 | 394.3 | 0 | 0.431 |
| Model 2 | 0.3218 |  | -0.348 | + | 3 | -194.984 | 396 | 1.68 | 0.186 |
| Model 3 | 0.1718 | + |  |  | 5 | -192.989 | 396 | 1.69 | 0.186 |

^1^ Rows represent candidate models with different combinations of predictors. When a continuous predictor is included in the model, a coefficient is provided. When a categorical predictor is included in the model, a plus sign (+) is provided.

^2^ Percent European wild boar ancestry calculated using the methods of Smyser et al. (2020).

^3^ Degrees of freedom of the model.

^4^ Log-likelihood of the model.

^5^ AIC of the model.

^6^ Δ AIC – difference between the AIC of the model and the AIC of the best (or top) model.

^7^ AIC weight of the model.

# Genomic inflation factors and Quantile-Quantile (QQ) plots

**Table S5.** Genomic inflation factors for each of the nine genome-wide association studies.

|  | **Genetic Model** | | |
| --- | --- | --- | --- |
| **Population^1^** | **Additive** | **Dominance** | **Recessive** |
| Comprehensive | 1.00 | 0.99 | 1.01 |
| HTS | 0.98 | 1.00 | 1.03 |
| CSHTS | 0.99 | 0.99 | 1.03 |

^1^ Study population examined: a comprehensive population consisting of adult feral swine throughout the invaded range within the contiguous United States (Comprehensive), a population of adult feral swine experiencing high PRV infection pressure with high temporal variability (HTS), and a population of adult feral swine experiencing temporally stable high PRV infection pressure (CSHTS).


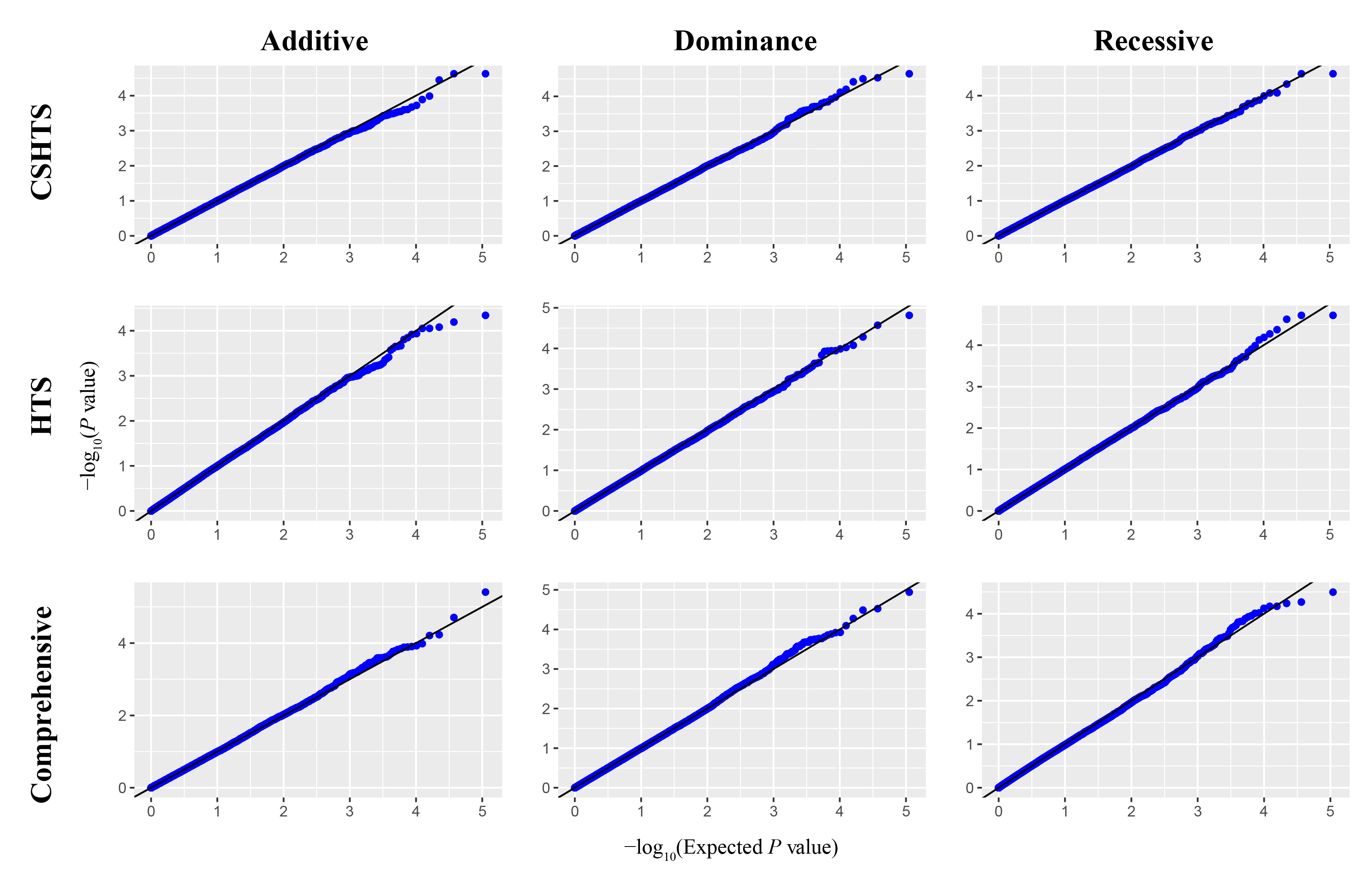
**Figure S1.** Quantile-quantile (QQ) plots for each of the nine genome-wide association studies. Three study populations: comprehensive study population (Comprehensive), high pseudorabies infection pressure with temporal variation study population (HTS), and high pseudorabies infection pressure without temporal variation study population (CSHTS). Three models of genetic architecture: additive, dominance, and recessive.

# Normalized enrichment score distributions


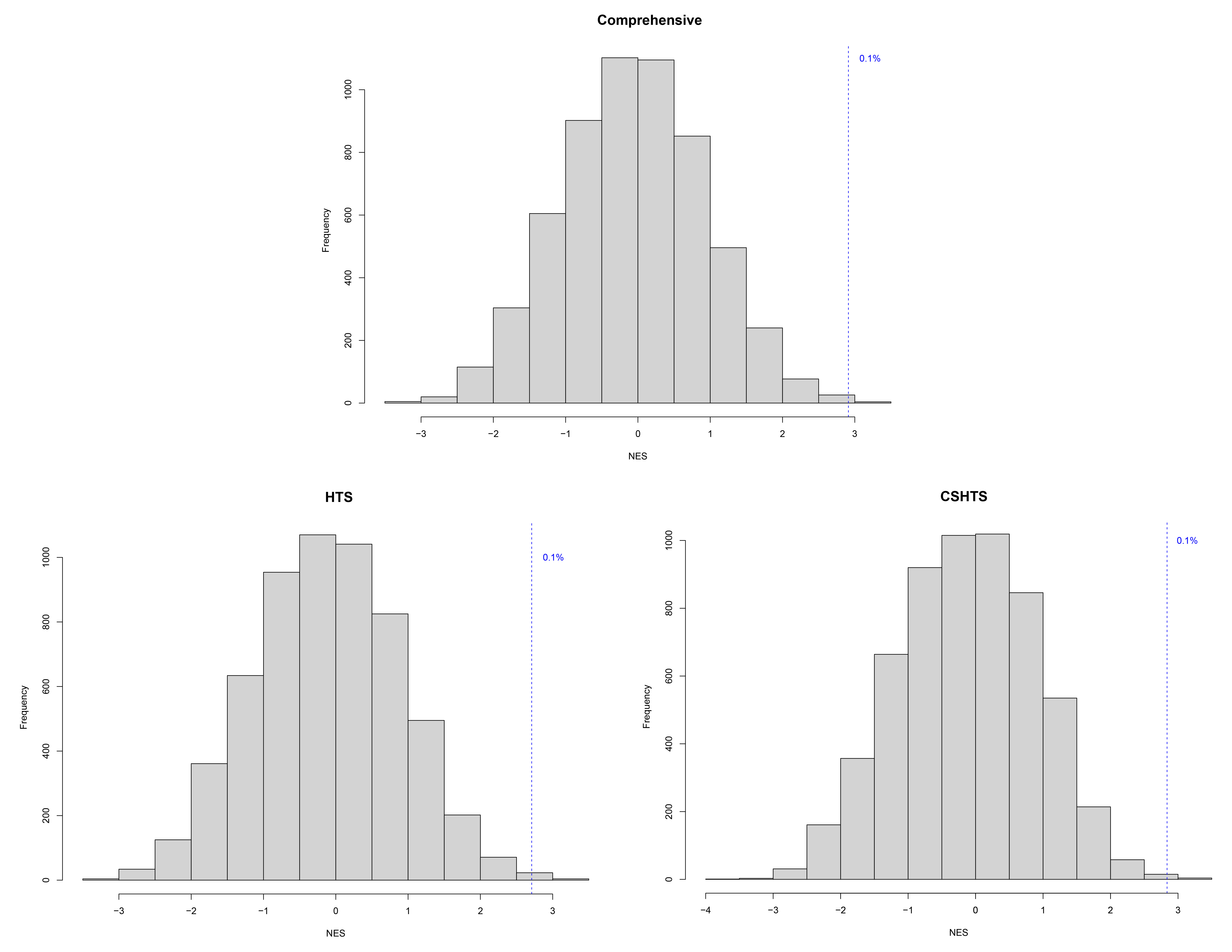
**Figure S2.** Histograms of normalized enrichment scores (NES) for each of the three study populations: comprehensive study population (Comprehensive), high pseudorabies infection pressure with temporal variation study population (HTS), and high pseudorabies infection pressure without temporal variation study population (CSHTS). The top 0.1% percentile of gene sets based on NES is represented by the vertical, dashed blue line.

# Leading edge genes

**Table S6.** Leading edge genes for gene sets associated with pseudorabies virus (PRV) infection status in adult feral (*Sus scrofa*) sampled across the invaded range in the contiguous United States using gene set enrichment analysis of single nucleotide polymorphism data (GSEA-SNP).

| **Gene Set**^1^ | ***n***^2^ | **LEGs**^3^ |
| --- | --- | --- |
| Muscle cell cellular homeostasis (GO:0046716) | 4 | *CAPN3, CAV3, CFL2, SGCZ* |
| Negative regulation of cellular protein localization (GO:1903828) | 36 | *ANGPT1, ASTN2, BAG3, BCL2L1, CAV3, CDT1, COMMD1, DAB2, DCLK1, DCLK2, DCLK3, DMTN, INPP5K, INSIG1, LEPROTL1,*  *MAPT, MIR155, MRAP, NF1, OTUD7B,*  *PARK7, PICALM, PID1, POLR1A,*  *RAB23, RIPOR2, ROCK2, SIAH3, SIRT4,*  *TAX1BP3, TMBIM1, TMEM59, TRIM40, UBAC2, UBE2G2, UBE2J1* |
| Spinal cord motor neuron differentiation (GO:0021522) | 6 | *GLI2, OLIG2, PAX6, PHOX2A, PTCH1, SOX4* |
| Ventral spinal cord development (GO:0021517) | 8 | *DAB1, GLI2, OLIG2, PAX6, PHOX2A, PTCH1, RELN, SOX4* |
| Protein localization to cilium (GO:0061512) | 15 | *ARL13B, BBIP1, BBS1, BBS9, CC2D2A, CCDC88A, DZIP1, EFCAB7, GGA1, IFT20, SNX10, TBC1D32, TUB, TULP4, WDR35* |
| Epithelial cell apoptotic process (GO:1904019) | 43 | *ABL1, ANGPT1, ANO6, BAD, BCL2L1, BID, BMPR2, BRAF, CAST, CCL2, CD248, COL18A1, COL4A3, DAB2IP, DNMT3A, E2F1, FASLG, FGA, FGB, GAS6, GATA2, ITGA4, KRT18, KRT8, MAP3K5, MIR132, MIR30B, MTOR, NFE2L2, PDX1, PIK3CG, PPARGC1A, RB1, RYR2, SCG2, SEMA5A, SFRP4, SIX3, TCF7L2, TERT, TNF, TNFAIP3, WFS2* |
| Membrane depolarization during cardiac muscle cell action potential (GO:0086012) | 8 | *ANK3, CACNA1C, CACNA1D, CACNB2, CAV3, RANGRF, SCN2B, SCN3B* |
| Cellular response to sterol (GO:0036315) | 11 | *AACS, ABCA1, GRAMD1B, HMGCS1, INSIG1, LRP8, MIR182, MIR96, PTCH1, RORA, RORC* |
| Sumoylation of transcription factors (Reactome) | 7 | *HIC1, MITF, PIAS1, SUMO2, SUMO3, TFAP2B,*  *TFAP2C* |
| Cell adhesion mediator activity (GO:0098631) | 27 | *ANXA1, BCAM, CNTN2, CNTN4, CNTN6, CXADR, DSCAM, DSCAML1, EMILIN1, EPCAM, ITGA11, ITGA2, KRT18, LRRC4C, NFASC, NTNG1, PALLD, PDLIM5, PKP2, RAB10, ROBO4, RPSA, S100A11, SIRPA, STXBP6, TACSTD2, TMOD3* |
| Negative regulation of synapse organization (GO:1905809) | 11 | *ARHGEF15, DNM3, EFNA1, FYN, GRIN2B, MIR30B, NGEF, PRNP, PTK2, ROBO2, SLIT1* |
| Glycosaminoglycan biosynthesis chondroitin sulfate (KEGG) | 11 | *B3GAT1, B3GAT3, CHST11, CHST15, CHSY1, CHSY3, CSGALNACT1, DSE, UST, XYLT1, XYLT2* |
| Regulation of calcium ion transport into cytosol (GO:0010524) | 26 | *ADCYAP1R1, AKAP6, ANK2, BCL2, CACNA1C, CAMK2D, CXCL10, CXCL11, CXCL9, DHRS7C, DIAPH1, DRD1, EPO, FYN, JPH2, NOS1, NPSR1, P2RY6, PLCG1, PRKCE, PRNP, PTPN6, RYR2, TMEM38B, TRPC3, UBASH3B* |
| Actin filament organization (GO:0007015) | 113 | *ACTN1, ACTN2, ACTN4, ACTR3B, ADD3, AP1AR, ARAP1, ARHGAP12, ARHGAP25, ARHGAP28, ARPC1A, ARPC1B, ARPC4, ASAP3, BCL2, CAPZA1, CAPZB, CARMIL1, CATIP, CD2AP, CDC42EP4, CDC42EP5, CFL2, CGNL1, CIT, CLASP1, CLASP2, COBL, CTNNA1, CTNNA2, CUL3, DIAPH1, DIAPH3, DLC1, DLG1, DPYSL3, EPHA1, F2RL1, FAM107A, FCHSD1, FCHSD2, FER, FHDC1, FHOD3, GMFB, GPR65, GRB2, HIP1, INPP5K, IQGAP2, LCP1, LIMA1, LIMCH1, MICAL1, MICAL2, MIR214, MSRB2, MTPN, MYO1B, MYO1C, MYOM1, NCKAP1, NEBL, NEDD9, NOX4, PAK1, PDXP, PHACTR1, PPARGC1B, PPFIA1, PPP1R9B, PRKCE, PRKN, PXN, RGCC, RHOA, RHOB, RHOBTB2, RHOBTB3, RHOC, RHOD, RHOU, RND1, ROCK2, S100A10, S1PR1, SAMD14, SEMA5A, SERPINF2, SH3BP1, SLIT2, SNX9, SORBS1, SORBS2, SPIRE1, SPIRE2, SPTA1, SSH1, SWAP70, TESK1, TMOD1, TMOD2, TNFAIP1, TPM2, TRIOBP, TRPV4, TSC1, TWF2, VILL, WASF3, WIPF2, WIPF3, ZYX* |
| Pyruvate metabolism and citric acid (TCA) cycle (Reactome) | 23 | *ACO2, CS, FAHD1, FH, GLO1, GSTZ1, LDHA, LDHB, LDHC, MDH2, ME1, ME2, ME3, MPC1, OGDH, PDHX, PDK1, PDK2, PDK4, PDPR, SDHC, SLC16A1, SUCLA2* |
| Thioester metabolic process (GO:0035383) | 17 | *ACACA, ACACB, ACBD6, ACOT11, ACOT8, ACSF2, ACSM2B, ACSM4, ACSM5, DGAT1, HMGCL, HSD17B4, MVK, OGDH, OXSM, SUCLA2, THEM5* |
| Sphingolipid metabolic process (GO:0006665) | 21 | *ASAH1, ESYT1, ESYT2, GALC, GBA2, GLTP, GM2A, KDSR, NEU3, NSMAF, ORMDL1, ORMDL2, PSAP, SERINC3, SGPL1, SGPP2, SMPD2, SPHK1, ST6GALNAC3, SUMF2, UGT8* |

^1^ Name of gene set and database it was derived from. Gene Ontology (GO), Kyoto Encyclopedia of Genes and Genome (KEGG), Reactome.

^2^ Number of leading edge genes in the gene set.

^3^ Leading edge genes in the gene set.
